# Medical and Philosophical Intelligence

**Published:** 1811-03

**Authors:** 


### MEDICAL AND PHILOSOPHICAL INTELLIGENCE.

Royal Society.—The Society, on meeting after the holidays, concluded the reading of Dr. Parry's paper on the means of curing certain nervous affections merely by pressure on the carotid artery; a method which he proposed several years ago, and has found effectual in almost every case.

The same evening a mathematical paper on the hyperbola was laid before the society by the Rev. Mr. Helens; but it was of a nature not to be read.

A curious account of a child born in Wales without eyes, or rather without eye-balls, was communicated by Mr. Jones: a small round white ball is found in the place of the eye, and the tunica conjunctiva was perfect. The mother attributes this organic defect to a fright which she received when seven months gone with child.

A letter from Dr. Wollaston to Dr. Marcet was read, in which Dr. W. related his experiments formerly made with a view to ascertain the existence of sugar in the serum of the blood of diabetic patients. The result of a considerable number of experiments, as well well as the imperfect attempts of Mr. Cruikshank and Dr. Rollo, convinced him of the non-existence of sugar in such serum. Dr. Marcet, at the instance of Dr. Wollaston, also made some experiments with the same view, and administered five grains of prussiat of potash (without danger) to a patient whose urine yielded a blue colour on the addition of iron.

On the 17th and 24th, a long paper by Mr. Macartney was read on the nature of vital heat. Mr. M. related the appearances exhibited in a great number of experiments on eggs and rabbits, made with a view to ascertain the origin and progress of animal heat in the young chick, &c. and from them concluded, that vital heat does not depend on respiration, and that it may exist in any form of matter. This conclusion, however, he expressed with extreme diffidence; and, as if either afraid of its validity or of the accuracy of his own experiments, partly wished to decline giving any opinion on a subject which he still deemed very complex and imperfectly understood.

---

*The National Vaccine Establishment* is about to circulate the following letter, addressed to the Director, Manager, or Superintendant of Public Charities, &c. &c. &c.

*National Vaccine Establishmeut,*
*Board Room, Leicester Square, Feb.* 14, 1811.

Sir,—This establishment has been directed by Government to extend the benefits of vaccination to all parts of the British dominions, and I am instructed by the Board to submit to your judgment the propriety and humanity of procuring the adoption of this Innocent Preventive of Small Pox among the children of
and among the poor in general.

Since vaccination has been generally employed in the nurseries of the nobility, gentry, and more informed class of society, the mortality occasioned by small pox has been confined to the poor.

In behalf of this description of persons, many of whom, from prejudice, are induced to reject the benefit offered to them; I have the honour to address you at the instance of the Board, and to lay before you the examples of some Charities, in which Vaccination has been successfully adopted.

In the Royal Military Asylum for the Children of Soldiers, where between eleven and twelve hundred children are now received, Vaccination has been practised from the commencement of the establishment in the year 1803; from that period to the present time, but one instance of death from small pox has occurred, and it is worthy of remark, that the individual who suffered had not undergone Vaccination, in consequence of a declaration of the mother that the child had passed through the small pox in infancy.

Vaccination was introduced into the Foundling Hospital in the year 1801, and every infant, soon after its admission, has since that period been vaccinated. From the commencement of this practice to the present time, no death has occurred from small pox, and in no instance instance has the preventive power of vaccination been discredited; although many children, as a test of its efficacy, have been repeatedly inoculated with the matter of small pox, and exposed to the influence of its contagion.

I am also directed by the Board to acquaint you, that in their opinion, the local and constitutional maladies which frequently follow the small pox, rarely, if ever succeed to the vaccine inoculation; that it produces neither peculiar eruptions nor new disorders of any kind, and they wish to call your attention to the fact ascertained by the Society for teaching the indigent Blind, namely, that nearly one fourth of the persons admitted into that charity have been deprived of their sight in consequence of small pox.

The Board have been induced to submit the above observations to your notice, on account of the number of deaths by small pox announced in the Bills of Mortality of the year 1810, which they are persuaded have arisen from the contagion propagated by persons of the poorer class, who from prejudice and unfounded alarms, have been induced to use small pox inoculation in preference to vaccination.

The Board have directed me to assure you that they disclaim every idea of interfering with the concerns of the Charity, but they have confidence that in the event of their suggestion meeting with the approbation of the Committee, the best and wisest plans will be adopted by that Body, for the public advantage.

I have the honor to be, Sir,
Your obedient humble Servant,
JA. HERVEY,
Register.

---

A singular case of derangement of structure in a fœtus, in consequence of original malformation, has lately occurred to Dr. Merriman. The mother had scarcely completed her eighth month, the fœtus was born alive, but died in about two hours; it was of the male sex; the penis was flattened and imperforate, and there were no traces of testicles; the rectum likewise was imperforate: but what particularly excited attention was an immense enlargement of the belly, evidently formed by a tumour within the abdomen, extending far above the navel. Dr. Merriman being very desirous of ascertaining what could produce this unusual appearance, obtained the consent of the friends to have this child opened, which was accordingly done the same evening by Mr. *Howship.*

On cutting into the abdomen, the tumour was found to consist of the bladder and ureters immensely enlarged and distended with urine; the ureters were become tortuous, and pouches were formed in them equalling in size the bladder at birth. The quantity of urine contained in the bladder and ureters exceeded half a pint, which is perhaps ten or twelve times as much as the bladder of a full grown fœtus generally contains. The kidnies were nearly covered with hydatids. This curious preparation is preserved in Mr. Heaviside's very valuable Museum.

MR.

Mr. George Singer, a gentleman of extensive acquirement in the various branches of experimental philosophy, and of most assiduous application to the objects of his particular pursuit, is now delivering a Course of Lectures on *Electrical, and Electro-Chemical Science*. The comprehensive scope of these Lectures, is dintinctly shewn by the subsequent statement.

*The First Part,* comprizing the History and Practice of Electricity, will give the origin and progress of Electrical Discovery, an experimental illustration of the principal historical facts; by which the epochs of the science are marked, and impressed on the memory by an appeal to the senses. An investigation of the Nature of Electrical Phenomena, in which an interesting diversity of experiments will be exhibited, and the Laws of Electrical Action investigated, and explained, on principles at once competent, intelligible, and consistent. In this part of the course some original experiments on the excitation of electrical effects, and on the variable appearance of charged surfaces will be detailed; the structure and management of the apparatus described; and instructions given for the successful prosecution of electrical inquiries.

*The Second Part,* on the natural Agencies of Electricity, will trace the influence of electricity in the production of various important phenomena of nature; connected with meteorology, vegetation, geology, and animal life. It will include a very full explanation of the nature and cause of thunder and lightning, in all its variety of effect; an account of the means devised for the protection of buildings, and ships, from injury by thunder storms; an investigation of the merits of the various means proposed, with directions for the most perfect insurance of safety; instructions for personal security, and for the defence of carriages, &c. &c. The influence of electricity in the production of meteors, falling stars, and other atmospheric phenomena, will be next examined and illustrated by a variety of experiments. The existence of water in the atmosphere, and its connection with electrical appearances will be also demonstrated, and applied to the explanation of some important natural arrangements. The experiments and inquiries on the connection of electricity with the functions of vegetable and animal life, will be also considered, and the opinions advanced on this subject examined. The facts which prove the active existence of electric powers in the animal economy, will be illustrated by a description of the Torpedo, Gymnotus, and other electric animals. The means by which these phenomena are produced being next considered, will lead to an investigation of the experiments of Galvani, and others, on the excitation of muscular motion, by electrical powers; these experiments will be illustrated by some striking phenomena, and the theories advanced to account for them will be fully explained.

*The Third Part,* on the Chemical Agencies of Electricity, is intended to exhibit a view of the rise and progress of that connection of electricity with chemical inquiry, which has contributed so rapidly to the improvement of the latter science. The experiments of Franklin, Priestley, Cavendish, Cuthbertson, and Morgan, constitute the first epoch, and are eminent for their influence in the propagation of chemical inquiry. The circumstances that led to the discovery of the voltaic battery; the invention of this instrument; and the discovery of its chemical agency, are are the next objects of consideration; and here the names of Nicholson, Henry, Cruickshank, and Davy claim the highest attention. The discovery that different chemical bodies had a tendency tc arrange themselves at the opposite surfaces of the battery; the opinion that these substances were formed by its influence; and the experiments of Mr. Davy in contradiction of this opinion, lead to the consideration of that brilliant series of discoveries which have so recently enriched and diversified chemical philosophy. The decompositions effected, the means of Analysis discovered, and the prospect of discovery opened, by these researches, will be attentively considered end exemplified by original observations and experiments.

*The Fourth, and concluding Part* will contain an examination of the present state of theoretical knowledge in the science of Electricity.

This part of the series is intended to render the exposition of the subject as complete as possible. It is presumed it will at the same time effect another important purpose; that of impressing on the memory the principal facts of the Science; as they will be recalled to the attention very powerfully, during the investigation of the most distinguished theories. The enquiry will be rendered interesting by the introduction of a variety of original experiments, some of which have been made in pursuance of *Mr. De Luc's* recent Analysis of the Voltaic Instrument. This analysis will be amply considered, as will also every other opinion of importance on the same subject.

To the extensive developement of the phenomena of the electric fluid, which this promises, we can add that Mr. Singer is particularly happy in illustrating the various opinions and theories, by the correctness and success of his experiments. We recommend this course of evening lectures to the medical student, not only as an ornamental branch of study, but as elucidating many laws of nature connected with MEDICAL SCIENCE.

---

### *On the Treatment of Pulmonary Inflammation in Horses; on Inoculating Glanders and Farcy.*

We are informed, on good authority, that Mr Colman, of the Veterinary College, has adopted a plan, successfully, of treating Horses labouring under inflammatory affections of the lungs, and coughs, viz. that of exposing them to the open, fresh, and cold air, in place of keeping them in warm stables.

Also the experiments of transferring the blood of glandered horses into the ass have been repeated, by which the glanders was produced; and again, of inoculating the horse with the matter of the nose of the glandered ass, and with the matter of farcy, by which the glanders was excited. Likewise the glandered horse became affected with the farcy.

---

### *On the cause of the Red Colour of the Blood.*

No fact seems to be more positively ascertained, by many respectable physiologists, than that the red colour of the blood is owing to red oxide of iron: there are, however, many others of equal authority, who do not allow that the asserted fact is proved. I shall be glad if the evidence on this point be further examined, and in the mean time offer the following experiment and observation.

*Experiment*—I collected a quantity of the red part of human blood by repeated ablutions of the crassamentum, by which I separated, of course, the coagulated lymph or albumen, and obtained the part desired in the form of a deposit from the water. This deposit was evaporated to dryness. It weighed 110 grains. By burning in a platina crucible, it afforded a half-fused, brown, tasteless substance in weight two grains and a half. It was boiled in muriatic acid, which dissolved part of it. On evaporating the solution duly, it was not found styptic to the taste; on the addition of tincture of gall-nut it became blackish; on the addition of prussiate of potash it afforded a deep blue coloured precipitate, which on caculation, however, did not yield above one half of a grain of reddish-brown powder. Now, by estimation, this half a grain of reddish-brown powder was obtained from 10,000, or twenty ounces of blood. Is it then probable that this quantity of blood should derive its colour from half a grain of iron.

*Observation*.—On exposing the crassamentum of blood to heat to separate the water from it, as soon as it grows hot, the colour changes from black, or red, to brown or grey. Is it probable that this change from *red*, or black, to *brown*, would occur, if iron oxide was the colouring matter of blood? *Edin. Jour.*

---

Dr. Curry, of Guy's Hospital, has just put to press the first volume of his promised work, "On the Nature of the Hepatic Function, the Purposes it serves in the Animal Œconomy, and the powerful Influence which a disorded state of it exerts, in exciting, aggravating, and modifying various Forms of Disease, both general and local."—It is expected to be comprised in two volumes, 8vo.

---

Mr. James Perry will shortly publish, in large quarto, "*Conchology*, or a History of Shells:" illustrated by more than 400 specimens, engraved the natural size of the shells, and coloured.

